# NLRP3 Inflammasome Promotes Myocardial Remodeling During Diet-Induced Obesity

**DOI:** 10.3389/fimmu.2019.01621

**Published:** 2019-07-16

**Authors:** Marina Sokolova, Ivar Sjaastad, Mieke C. Louwe, Katrine Alfsnes, Jan Magnus Aronsen, Lili Zhang, Solveig B. Haugstad, Bård Andre Bendiksen, Jonas Øgaard, Marte Bliksøen, Egil Lien, Rolf K. Berge, Pål Aukrust, Trine Ranheim, Arne Yndestad

**Affiliations:** ^1^Research Institute of Internal Medicine, Oslo University Hospital, Rikshospitalet, Oslo, Norway; ^2^Faculty of Medicine, Institute of Clinical Medicine, University of Oslo, Oslo, Norway; ^3^Center for Heart Failure Research, University of Oslo, Oslo, Norway; ^4^K.G. Jebsen Center for Cardiac Research, University of Oslo, Oslo, Norway; ^5^Institute for Experimental Medical Research, Oslo University Hospital Ullevål, Oslo, Norway; ^6^Bjørknes College, Oslo, Norway; ^7^Division of Infectious Diseases and Immunology, University of Massachusetts Medical School, Worcester, MA, United States; ^8^Centre of Molecular Inflammation Research, Norwegian University of Science and Technology (NTNU), Trondheim, Norway; ^9^Department of Clinical Science, Department of Heart Disease, Haukeland University Hospital, University of Bergen, Bergen, Norway; ^10^Section of Clinical Immunology and Infectious Diseases, Oslo University Hospital Rikshospitalet, Oslo, Norway

**Keywords:** inflammasome, NLRP3, heart, cardiac remodeling, obesity, high-fat diet

## Abstract

**Background:** Obesity is an increasingly prevalent metabolic disorder in the modern world and is associated with structural and functional changes in the heart. The NLRP3 inflammasome is an innate immune sensor that can be activated in response to endogenous danger signals and triggers activation of interleukin (IL)-1β and IL-18. Increasing evidence points to the involvement of the NLRP3 inflammasome in obesity-induced inflammation and insulin resistance, and we hypothesized that it also could play a role in the development of obesity induced cardiac alterations.

**Methods and Results:** WT, *Nlrp3*^−/−^, and *ASC*^−/−^ (*Pycard*^−/−^) male mice were exposed to high fat diet (HFD; 60 cal% fat) or control diet for 52 weeks. Cardiac structure and function were evaluated by echocardiography and magnetic resonance imaging, respectively. Whereas, NLRP3 and ASC deficiency did not affect the cardiac hypertrophic response to obesity, it was preventive against left ventricle concentric remodeling and impairment of diastolic function. Furthermore, whereas NLRP3 and ASC deficiency attenuated systemic inflammation in HFD fed mice; long-term HFD did not induce significant cardiac fibrosis or inflammation, suggesting that the beneficial effects of NLRP3 inflammasome deficiency on myocardial remodeling at least partly reflect systemic mechanisms. *Nlrp3* and *ASC* (*Pycard*) deficient mice were also protected against obesity-induced systemic metabolic dysregulation, as well as lipid accumulation and impaired insulin signaling in hepatic and cardiac tissues.

**Conclusions:** Our data indicate that the NLRP3 inflammasome modulates cardiac concentric remodeling in obesity through effects on systemic inflammation and metabolic disturbances, with effect on insulin signaling as a potential mediator within the myocardium.

## Introduction

Obesity is an emerging health problem in the modern world, leading to a reduced life expectancy, and is defined as increased adipose mass resulting from a chronic imbalance between energy intake and expenditure (1). Obesity-related conditions, such as insulin resistance, type 2 diabetes mellitus (T2DM), cardiovascular disorders (CVD), non-alcoholic fatty liver disease (NAFLD) are a great concern, in particular in developing countries, but the mechanisms by which obesity promotes these disorders still remains unclear (2, 3).

Abundant evidence suggests that obesity is accompanied by structural and functional alterations in the heart (4–6). According to a recently established paradigm regarding the impact of obesity on the cardiac geometry, obesity is associated with left ventricle (LV) hypertrophy with predominance of concentric remodeling (4–6). The majority of published studies have concluded that obesity results in subclinical impairment of LV systolic and diastolic functions, which are believed to be precursors to more overt forms of cardiac dysfunction and heart failure (HF). Several theories have been postulated to explain these obesity-associated cardiac abnormalities, such as alterations in myocardial substrate utilization, mitochondrial dysfunction, neurohormonal dysfunction, leptin resistance, and impaired insulin signaling (4, 7–10). However, the multifaceted interplay between direct cardiac effects of obesity and its associated comorbidities (i.e., T2DM and atherosclerosis) that also impact the myocardium makes it challenging to understand the relation between obesity and different aspects of cardiac remodeling.

The cytosolic pattern recognition receptor NLRP3 is an important part of the innate immune system that can sense danger signals from various microbes, as well as non-microbial endogenous signals, such as extracellular ATP, crystals, saturated fatty acids (FA) and certain other metabolic stress-related molecules (11–14). Upon activation, that occurs in a two-step manner, NLRP3 forms a multiprotein complex called NLRP3 inflammasome with the adaptor protein ASC, also referred to as Pycard, resulting in caspase-1 activation. Active caspase-1 cleaves the pro-forms of the cytokines interleukin (IL)-1β and IL-18 to their active and secreted forms of which IL-1β is of particular importance as an upstream mediator in the inflammatory cytokine cascade (15). Compelling evidence suggests a significant role of NLRP3 inflammasome in the initiation and progression of metaflammation (i.e., metabolically-induced inflammation) and related diseases, such as obesity, T2DM, NAFLD, and atherosclerosis (16–18). In addition, we and others have recently demonstrated that NLRP3 inflammasome is functional in the heart with the potential to regulate cardiac function and cell death (19). Based on these previous studies, we hypothesized that the NLRP3 inflammasome plays a role in the development of cardiac dysfunction and remodeling during diet-induced obesity. The present study was designed to explore this hypothesis by examining *Nlrp3* and *ASC* (*Pycard*) deficient mice in a model of obesity induced cardiac remodeling.

## Methods

### Mice

C57BL/6J mice were purchased from The Jackson Laboratory (Bar Harbor, ME, USA). All knockout mice were back-bred onto the C57BL/6 strain. *Nlrp3*^−/−^ and *Asc*^−/−^ (*Pycard*^−/−^) mice were generated by Millenium Pharmaceuticals (Cambridge, MA, USA), back-bred at least seven (*Nlrp3*^−/−^) or nine (*Asc*^−/−^/ *Pycard*^−/−^) generations before being used (20, 21). Mice were housed in an air-conditioned, temperature-regulated room with a 12/12 h daylight/night cycle with free access to water and food. The diet and genetic background are major determinants of gut microbial composition which again could influence metabolic and inflammatory diseases. To minimize the effects of other factors than genetics in our study, including effects on gut microbiota, the mice were co-housed throughout the study. The separate mouse strains were littermates, bred from the same parents, raised in the same cage until weaning where 4–6 mice of the same strain where co-housed in the same open cages (Eurostandard type III), and all cages were placed in the same room in a randomized manner. Obesity was induced by feeding mice a HFD (D12492), composed of 60% fat, 20% protein, and 20% carbohydrate (Research Diets, New Brunswick, NJ, USA) for 52 weeks. Control mice were fed a low fat standardized control diet, containing 10% fat, 20% protein and 70% carbohydrate (D12450B, Research Diets). Body weight was regularly monitored weekly. Food intake was determined at 21 weeks by weighing the food and correcting for the amount not eaten, including spillage. The experimental animal protocol (FOTS id 4641) was approved by the Norwegian Animal Research Committee and conforms to the Guide for the Care and Use of Laboratory Animals published by the US National Institutes of Health (NIH Publication, 8th Edition, 2011).

### Echocardiography

Echocardiographic examination was performed with the VEVO 2100 system (VisualSonics, Toronto, Canada). Mice were lightly anesthetized with a mixture of 98.25% O_2_ and 1.75% isoflurane maintained by mask ventilation, and were placed on a heated examination table to maintain body temperature. Standard echocardiography examination, including long and short axis images of the LV and atrium, and doppler recordings, were performed (22). Recorded data were analyzed offline using the Vevo 2100 1.1.0 software (VisualSonics). Relative LV wall thickness was calculated with the formula: (IVSd + LVPWd)/LVIDd.

### Magnetic Resonance Imaging

Magnetic Resonance Imaging (MRI) experiments were performed by using a 9.4T preclinical MR system (Agilent Technologies, Inc., CA, USA). Two different gradients and RF coil set-ups have been employed because of the mice changing size during the study period. The images were acquired with either a 100 gauss/cm, 60 mm ID gradient and a quadrature volume RF coils (35 mm ID, Rapid Biomedical, DE) or a 60 gauss/cm, 72 mm ID gradient and an active-decoupled phase array surface coil (Neos Biotec, Spain) plus quadrature volume RF coils (72 mm ID, Rapid Biomedical) combination. Anesthesia was induced with a mixture of O_2_ and 4% isoflurane, and maintained with 1.5–2.0% isoflurane in freely breathing animals. Animal temperature was maintained around 37°C by heated air. Cardiac and respiratory gated cine-MRI was acquired in the true short-axis orientation. The key parameters were 1 mm slice thickness; TE/TR 2.2/4.6 ms; 2 averages; field of view 25.6 × 25.6 mm; matrix size 128 × 128. One mid-ventricular slice and one four chamber long axis slice were acquired using a nine-point velocity-encoded black-blood gradient echo cine sequence as previously described (23). Imaging parameters were: TE = 2.4 ms; TR = 3.2 ms; field-of-view = 30 × 30 mm or 40 × 40 mm; acquisition matrix 96 × 96 and zero filled to 128 × 128; slice thickness 1 mm; venc = 10 cm/s, averages = 2 (using rotating field-of-view). Data were analyzed as previously described (24).

### Blood and Tissue Sampling

Mice were fasted for 4 h and put in deep anesthesia with a mixture of 4–5% isoflurane and O_2_. Arterial blood was collected (by a small incision of the carotid artery) into tubes containing 50 μl of 0.5 M EDTA. Plasma was prepared by centrifugation at 500 × g for 20 min and 4°C, snap-frozen in liquid N_2_ and stored at −80°C. The heart was extirpated and separated into LV and RV, together with lungs and liver, rinsed in saline solution, blotted dry and weighed. A standardized 2 mm slice was taken from the LV using a mouse heart slicer matrix (Zivic instruments, Pittsburgh, PA, USA). The heart slice and the left lateral lobe of the liver were fixated in 4% formalin and embedded in paraffin. Remaining tissue was snap-frozen in liquid N_2_ and stored at −80°C.

### Analysis of Glucose, Lipids, and Inflammatory Cytokines

Plasma insulin was determined by Mouse Ultrasensitive Insulin ELISA (ALPCO, Salem, NH, USA), plasma leptin by Mouse Leptin ELISA (ALPCO), plasma glucose by Mouse Glucose Colorimetric Assay kit (Cayman, Ann Arbor, MI, USA), triglycerides by LabAssay Triglyceride (WakoPure Chemical Industries, Richmond, VA, USA), and cholesterol by Cholesterol E (Wako Diagnostics, Richmond, VA, USA). Homeostasis model assessment—insulin resistance (HOMA-IR) was calculated using the following formula: HOMA-IR = [I_0_(mU/L) × G_0_(mg/dl)]/405 (25). Plasma levels of interleukin IL-18 and TNF were measured by using multiplex magnetic bead assay (Bio-Rad Laboratories, Berkeley, CA, USA) following the manufacturer's instructions. LV triglyceride levels were measured using LabAssay Triglyceride kit (WakoPure Chemical Industries).

### Liver Histology and Lipids

To examine liver histology, livers were fixed in formalin and embedded in paraffin and then cut into 5 μm sections. Sections were deparaffinized and stained with hematoxylin (Vector Laboratories, Burlingame, CA, USA) and eosin (Histolab Products AB, Gothenburg, Sweden). Images were captured by use of a Nikon DS Fi1 camera and a Nikon Eclipse E400 microscope (Nikon Instruments, Melville, NY, USA). Liver lipids were extracted from frozen samples according to Bligh and Dyer (26), evaporated under nitrogen, and re-dissolved in isopropanol before analysis. Lipids from liver were measured enzymatically on a Hitachi 917 system (Roche Diagnostics GmbH, Mannheim, Germany) using the TAG kit (Triglycerides GPO-PAP) and cholesterol kit (CHOD-PAP) from Roche Diagnostics.

### Cardiac Histology and Immunohistochemistry

Four micron transverse sections of formalin-fixed, paraffin-embedded mouse hearts were deparaffinized in xylene, rehydrated in alcohol series and immersed in distilled water, followed by high-temperature antigen retrieval in citrate buffer (pH 6) and blocked with 0.5% bovine serum albumin (Sigma-Aldrich, St. Louis, MO, USA). Slides were stained with primary antibody against Mac-2 (1:750, Cedarlane, Burlington, Canada) for 1 h at room temperature. After washing, slides were incubated for 30 min with peroxidase-conjugated secondary antibody (Impress-Vector, Vector Laboratories), rinsed and developed with chromogen for immunoperoxidase staining (DAB Plus, Vector Laboratories). The sections were counterstained with hematoxylin. Omission of the primary antibody was used as negative control. The stained sections were scanned (AxioScan Z1, Carl Zeiss, Oberkochen, Germany), and the amount of positive DAB-staining was quantitatively assessed using z9.uio.no, an in-house analysis application devised for whole slide images, by estimating cross sectional coverage of antibody expression within the tissue relative to the total area of the cross section of the tissue.

To measure cardiomyocyte (CM) cross-sectional area, LV sections were deparaffinized and rehydrated as described above and boiled at 98°C in citrate buffer pH 6, followed by 20 min cooling at room temperature (RT) before overnight incubation at (4°C) with Alexa-488 conjugated wheat germ agglutinin (WGA; Thermo Fisher Scientific, Waltham, MA, USA). Sections were rinsed in PBS and coverslipped with a water-soluble antifading mounting medium (Thermo Fisher Scientific). Areas showing CM cross sections were photographed with Nikon Eclipse E400 fluorescence microscope and CM area was quantified by an in house made macro for ImageJ (27). All histological analyses were performed blinded of genotype and treatment.

### Collagen Staining

For collagen staining, sections were deparaffinized and rehydrated as described above. Sections were thereafter incubated for 60 min in Sirius Red Solution (Histolab Products) followed by 2 × rinsing in acidified water containing 5% acetic acid. After two quick dips in 100% EtOH, the sections were incubated in xylene and mounted with Eukitt® (Sigma-Aldrich). The stained sections were scanned and analyzed as described for Mac-2, but detecting red chromogen instead of DAB-positive staining.

### Quantification of Gene Expression

Total RNA from mouse LV was extracted using TRIzol (Invitrogen, San Diego, CA, USA), DNase treated, cleaned up using RNeasy Mini Columns (Qiagen, Hilden, Germany), and stored at −80°C. cDNA was synthesized using High Capacity cDNA Reverse Transcription Kit (Thermo Fisher Scientific). Quantification of gene expression was performed in duplicate by quantitative real-time PCR, using Power Sybr Green Master Mix (Applied Biosystems, Foster By, CA, USA). Target gene expression was quantified using the relative standard curve method, using a standard curve generated with serial dilution of a pool of aliquots of sample cDNA, and subsequently normalized to glyceraldehyde 3-phosphate dehydrogenase (GAPDH) gene expression. Primers, designed to span exon-exon boundaries to avoid amplification of genomic DNA, were used. The sequences of the specific PCR-primers are listed in Supplementary Table S1.

### Assessment of Peripheral Insulin Sensitivity

WT and *Nlrp3*^−/−^ mice on HFD or control diet for 52 weeks were fasted for 4 h and received an i.p. injection of insulin (2 IU/kg of Novolin GE, Novo Nordisk, Bagsværd, Denmark). The mice were sacrificed 10 min after insulin administration, LV and liver samples were isolated and snap-frozen in liquid N_2_, and protein homogenates were prepared in T-PER Tissue Protein Extraction Reagent (Thermo Fisher Scientific) supplemented with protease and phosphate inhibitors (Complete Protease Inhibitor Cocktail, Roche Diagnostics), cleared by centrifugation, and concentrations were measured using a Pierce BCA Protein Assay (Thermo Fisher Scientific). Protein homogenates were separated under denaturing conditions on 10% SDS-polyacrylamide gels (Mini-PROTEAN TGX Precast gels, Bio-Rad, Hercules, CA, USA) and electro-blotted on to PVDF membranes (Thermo Fisher Scientific). The membranes were blocked in Superblock T20 (Thermo Fisher Scientific), and incubated with antibodies against phospho-Akt Ser473 (1:1,000 dilution; Cat# 9271, Cell Signaling Technology, Danvers, HA, USA), Akt (1:1,000; Cat# 9272, Cell Signaling Technology), and GAPDH (0.05 μg/ml; Cat# G8795, Sigma-Aldrich) which was used as a normalization control for the proteins of interest. Thereafter, the blots were incubated with a horseradish peroxidase-conjugated anti-rabbit or anti-mouse antibody (Cell Signaling Technology). Protein expression was detected by chemiluminescence (SuperSignal Dura; Thermo Fisher Scientific) and the Fujifilm LAS-3000 Imaging system. Densitometric quantification was performed using ImageJ.

### Statistical Analysis

GraphPad Prism 7.0 software was used for data analysis (GraphPad Software, CA, USA). Statistical analyses were performed using two-way ANOVA. Student *t*-tests were performed where two-way ANOVA was significant. *p*-value below 0.05 was considered as statistical significance. Data are shown as mean ± standard error of the mean (SEM).

## Results

### NLRP3 Inflammasome Does Not Affect Obesity-Induced LV Hypertrophy

**Figure 1:**
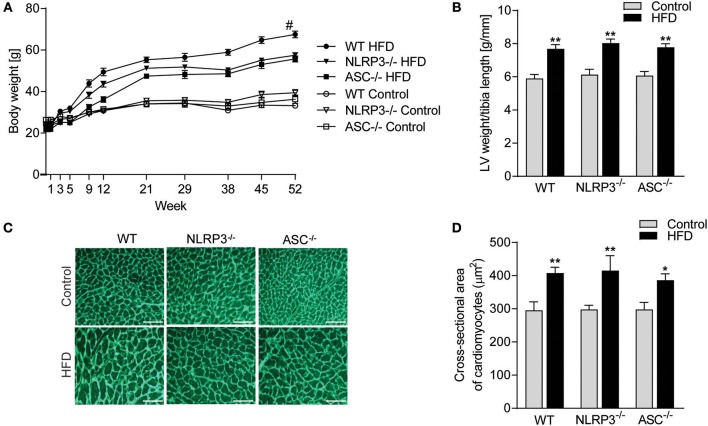
NLRP3 inflammasome does not affect obesity-induced LV hypertrophy. WT, *Nlrp3*^−/−^, and *Asc*^**−/−**^ (*Pycard*^−/−^) male mice were exposed to high fat diet (HFD; 60 cal% fat) or control diet for 52 weeks. **(A)** Comparison of bodyweight gain in mice on control or HFD during the 52 weeks [WT: Control, *n* = 12; HFD, *n* = 12, *Nlrp3*^−/−^: Control, *n* = 11; HFD, *n* = 12, and *Asc*^**−/−**^ (*Pycard*^−/−^): Control, *n* = 10; HFD, *n* = 12]. **(B)** Left ventricle (LV) weight normalized to tibia length weeks [WT: Control, *n* = 11; HFD, *n* = 11, *Nlrp3*^−/−^: Control, *n* = 8; HFD, *n* = 8, and *Asc*^**−/−**^ (*Pycard*^−/−^): Control, *n* = 10; HFD, *n* = 6]. **(C)** Representative images of wheat germ agglutinin (WGA) stained LV sections [WT: Control, *n* = 8; HFD, *n* = 9, *Nlrp3*^−/−^: Control, *n* = 7; HFD, *n* = 7, and *Asc*^**−/−**^ (*Pycard*^−/−^): Control, *n* = 8; HFD, *n* = 5). Scale bar: 100 μm. **(D)** Quantification of cardiomyocyte cross-sectional surface area. Data are means ± SEM. ^*^P < 0.05, ^**^P < 0.01 vs. control diet as determined by two-way ANOVA and Tukey's multiple comparisons test. ^#^*P* < 0.05 vs. NLRP3-HFD and ASC-HFD as determined by repeated measures two-way ANOVA and Tukey's multiple comparisons test.

Exposure to a HFD for 52 weeks induced profound obesity as compared to mice fed a control diet in all three genotypes (i.e., WT, *Nlrp3*^−/−^, and *As*c^−/−^ [*Pycard*^−/−^] mice) (Figure 1A). A significant separation of body weight between mice on HFD and control diet was observed from week 9, determining the initial moment of obesity. Notably, although all mouse genotypes showed increased weight during HFD, WT mice gained significantly more weight than the inflammasome deficient mice (Figure 1A). In contrast, no differences in weight gain were seen between the three genotypes during control diet (Figure 1A). Importantly, there were no significant differences in food intake comparing the different mouse genotypes (Supplementary Figure S1). As expected, HFD-induced obesity was associated with significant cardiac hypertrophy in WT mice, as indicated by an increased LV mass normalized to tibia length (Figure 1B) and increased CM cross-sectional area (Figures 1C,D). However, whereas NLRP3 and ASC deficiency affected body weight during HFD, it did not affect obesity-induced hypertrophic response (Figures 1B–D).

### NLRP3 and ASC Deficiency Have a Beneficial Effect on Obesity-Induced LV Remodeling and Dysfunction

**Figure 2:**
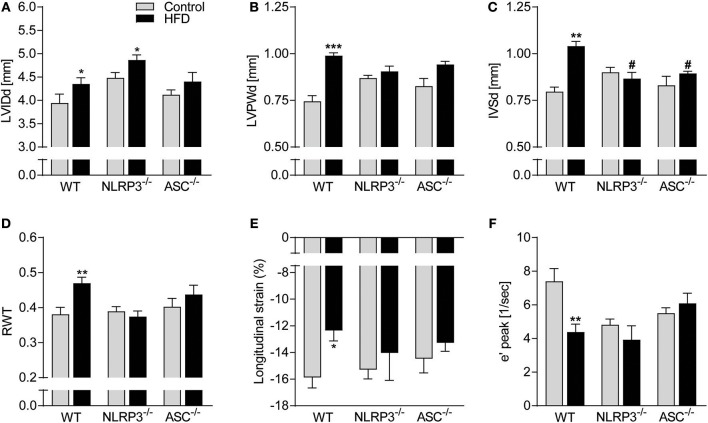
Beneficial effect of NLRP3 and ASC deficiency on obesity-induced LV concentric remodeling and dysfunction. WT, *Nlrp3*^−/−^, and *Asc*^**−/−**^ (*Pycard*^−/−^) male mice were exposed to a high fat diet (HFD; 60 cal% fat) or a control diet for 52 weeks. Cardiac structure and function was assessed by echocardiography. WT: Control, *n* = 6; HFD, *n* = 10, *Nlrp3*^−/−^: Control, *n* = 6; HFD, *n* = 7, and *Asc*^**−/−**^ (*Pycard*^−/−^): Control, *n* = 6; HFD, *n* = 7, and cardiac magnetic resonance imaging [MRI; WT: Control, *n* = 6; HFD, *n* = 6, *Nlrp3*^−/−^: Control, *n* = 6; HFD, *n* = 6, and *Asc*^**−/−**^ (*Pycard*^−/−^): Control, *n* = 6; HFD, *n* = 6]. **(A)** Left ventricular internal diameter at end diastole (LVIDd), **(B)** LV posterior wall thickness at end diastole (LVPWd), **(C)** interventricular septum thickness at end diastole (IVSd), **(D)** LV relative wall thickness (RWT), **(E)** longitudinal strain (MRI), and **(F)** early diastolic mitral annular velocity (e′ peak; MRI). Data are means ± SEM. ^*^P < 0.05, ^**^P < 0.01, ^***^P < 0.001 vs. control diet. ^#^*P* < 0.05 vs. WT HFD.

Cardiac structure was monitored by echocardiography. As displayed in Figure 2A, HFD induced a significant increase in LV end-diastolic internal diameter (LVIDd) in WT and *Nlrp3*^−/−^ mice, with a non-significant trend in *Asc*^**−/−**^ (*Pycard*^−/−^) mice (*p* = 0.22). WT mice on HFD also had markedly increased LV end-diastolic posterior wall (LVPWd) thicknesses and end-diastolic intraventricular septum (IVSd) (Figures 2B,C), but, notably, this was not observed in *Nlrp3*^−/−^ and *Asc*^**−/−**^ (*Pycard*^−/−^) mice. To further assess geometrical changes in the LV, we calculated relative wall thickness (RWT). This revealed a clear pattern of concentric remodeling in obese WT mice (RWT > 0.48), but not in *Nlrp3*^−/−^ and *Asc*^**−/−**^ (*Pycard*^−/−^) mice, which were protected against these obesity-induced alterations (Figure 2D).

Cardiac MRI analysis allowed us to more accurately assess cardiac mechanics and function and showed that obesity induced a significant reduction in LV systolic function in WT mice, but not in *Nlrp3*^−/−^ and *Asc*^**−/−**^ (*Pycard*^−/−^), as determined by longitudinal strain (Figure 2E). Moreover, whereas obese WT mice displayed impaired LV diastolic function as evident by a markedly reduced mitral annulus velocity during early diastole (e′), this was not seen in *NLRP3* and *ASC* (*Pycard*) deficient mice (Figure 2F). Notably, on control diet both *Nlrp3*^−/−^ and *Asc*^**−/−**^(*Pycard*^−/−^) mice had reduced e′ compared to WT, but obesity did not affect e′ in these mice. There were no statistical differences in gene expression of atrial natriuretic peptide (ANP) between the different groups, indicating that the fetal gene programme was not activated by HFD (Supplementary Figure S2).

Taken together, our results so far show that NLRP3 and ASC deficiency during obesity does not affect the hypertrophic response but prevents obesity-induced LV concentric remodeling and early signs of systolic and diastolic dysfunction.

### NLRP3 and ASC Deficiency Protects Against Obesity-Induced Metabolic Dysfunction and Inflammation

**Figure 3:**
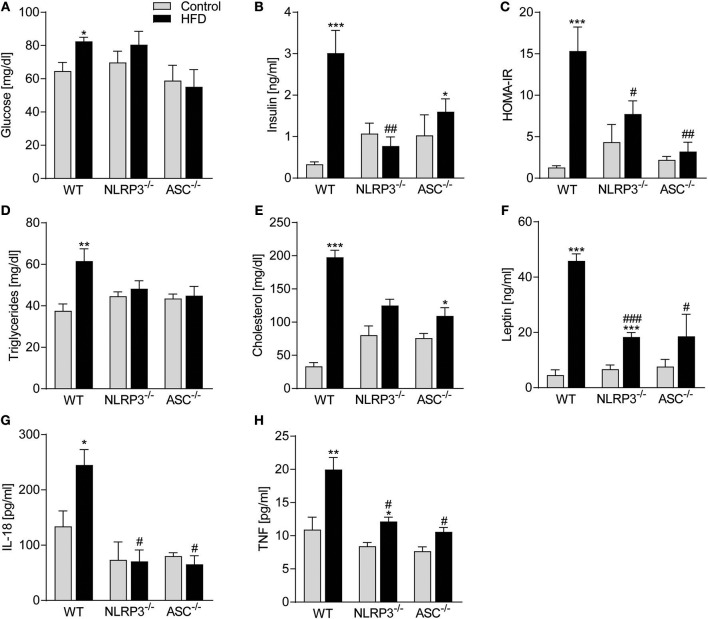
NLRP3 and ASC deficiency protects mice against obesity-induced metabolic dysregulation and inflammation. WT, *Nlrp3*^−/−^, and *Asc*^**−/−**^ (*Pycard*^−/−^) male mice were exposed to a high fat diet (HFD; 60 cal% fat) or a control diet for 52 weeks and plasma was collected. Plasma **(A)** glucose, **(B)** insulin, **(C)** HOMA-IR [homeostatic model assessment of insulin resistance calculated from measured glucose and insulin levels], **(D)** triglycerides, **(E)** cholesterol, **(F)** leptin, **(G)** interleukin (IL)-18, and **(H)** tumor necrosis factor (TNF). WT: Control, *n* = 8; HFD, *n* = 8, *Nlrp3*^−/−^: Control, *n* = 8; HFD, *n* = 7, and *Asc*^−/−^: Control, *n* = 7; HFD, *n* = 7. Data are means ± SEM. ^*^P < 0.05, ^**^P < 0.01, ^***^P < 0.001 vs. control diet; ^#^*P* < 0.05 vs. WT-HFD as determined by two-way ANOVA and Tukey's multiple comparisons test.

Obesity-induced changes in the myocardium could be secondary to systemic changes. We, therefore, next examined the influence of NLRP3 inflammasome on systemic, metabolic and inflammatory changes following 52 weeks on HFD. Plasma glucose and in particular insulin levels and insulin resistance, as assessed by HOMA-IR, were all elevated in obese WT mice (Figures 3A–C), with no significant changes in *Nlrp3*^−/−^ and *Asc*^**−/−**^ (*Pycard*^−/−^) mice. This suggests that the absence of inflammasome components led to improved maintenance of glucose homeostasis and increased insulin sensitivity during HFD. Similarly, *Nlrp3*^−/−^ and *Asc*^**−/−**^ (*Pycard*^−/−^) mice were protected against HFD-induced dyslipidaemia, while WT mice demonstrated significantly elevated triglycerides (TG) and cholesterol plasma levels in response to HFD (Figures 3D,E). Excessive leptin production is associated with high BMI and insulin resistance in T2DM (28), and as expected, HFD-fed WT mice exhibited a marked increase in plasma leptin compared with mice on control diet (Figure 3F). This increase in leptin levels during HFD was significantly attenuated in *NLRP3* and *ASC* (*Pycard*) deficient mice (Figure 3F). Finally, metabolic disturbances during obesity seems to interact with low-grade inflammation, and as shown in Figure 3G, IL-18 levels were significantly elevated in obese WT mice, but remained unchanged in HFD fed *Nlrp3*^−/−^ and *Asc*^**−/−**^ (*Pycard*^−/−^) mice (Figure 3G), using an assay which detects only the mature form of IL-18. Furthermore, HFD increased plasma levels of TNF, which was alleviated by NLRP3 and ASC deficiency (Figure 3H). Thus, it seems that NLRP3 and ASC deficiency protects mice against HFD-induced metabolic dysregulation, increased leptin levels and systemic inflammation. Plasma levels of IL-1β were under the detection limit in all mice (data not shown), using a highly sensitive method (Bio-Plex Pro, Bio-Rad).

### Absence of NLRP3 Inflammasome Components Suppresses Obesity-Induced Hepatic Steatosis

**Figure 4:**
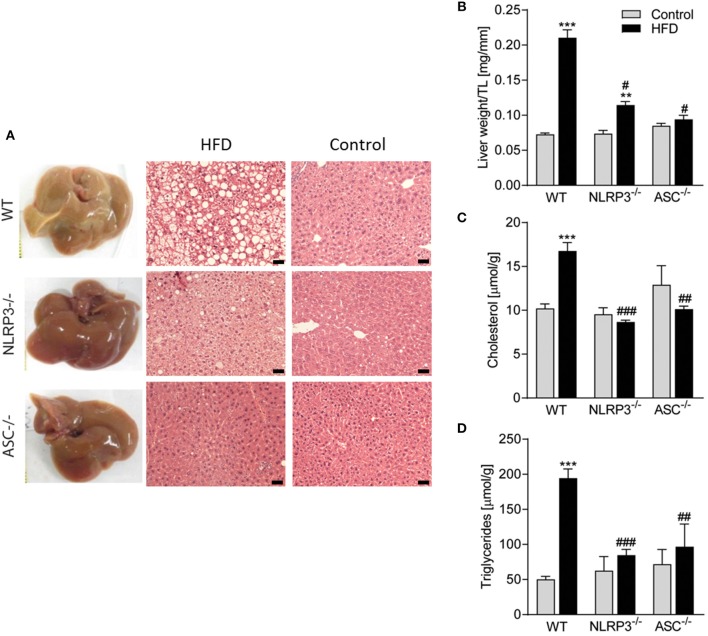
Obesity-induced hepatic steatosis is suppressed by NLRP3 and ASC deficiency. WT, *Nlrp3*^−/−^, and *Asc*^**−/−**^ (*Pycard*^−/−^) male mice were exposed to high fat diet (HFD; 60 cal% fat) or a control diet for 52 weeks and liver was extirpated. **(A)** Representative images of livers and hematoxylin and eosin staining from mice on HFD and Control diet. Scale bar: 100 μm. **(B)** Liver weights related to tibia length (TL). Hepatic levels of **(C)** cholesterol and **(D)** triglycerides. WT: Control, *n* = 10; HFD, *n* = 10, *Nlrp3*^−/−^: Control, *n* = 7; HFD, *n* = 7, and *Asc*^**−/−**^(*Pycard*^−/−^): Control, *n* = 9; HFD, *n* = 5. Data are shown as mean ± SEM. ^**^P < 0.01, ^***^P < 0.001 vs. control diet; ^#^*P* < 0.05, ^##^*P* < 0.01, ^###^*P* < 0.001 vs. WT-HFD as determined by two-way ANOVA and Tukey's multiple comparisons test.

Liver is a target organ for metabolic changes during obesity. We, therefore, performed a thorough pathological examination of the liver with respect to mouse genotypes and the diets. As illustrated in Figure 4A, consumption of a HFD resulted in an abnormal appearance with atypical yellowish coloration of the livers from WT mice with severe steatosis and lipid droplet accumulation, while livers from *Nlrp3*^−/−^ and *Asc*^**−/−**^ (*Pycard*^−/−^) mice retained dark red coloration without steatosis. There was no accumulation of lipid droplets in livers from the mice on a control diet in either of the genotypes as evaluated by hematoxylin and eosin staining (Figure 4A). Liver weights were markedly elevated in WT mice compared with *Nlrp3*^−/−^ and *Asc*^**−/−**^ (*Pycard*^−/−^) mice during HFD, but not during control diet (Figure 4B). HFD-induced liver steatosis in WT mice was also verified biochemically with markedly elevated hepatic levels of cholesterol and TG (Figures 4C,D), and importantly, *Nlrp3*^−/−^ and *Asc*^**−/−**^ (*Pycard*^−/−^) livers showed significantly reduced levels of these lipid components in response to HFD feeding compared with WT mice (Figures 4A–D). Thus, in line with our findings that *NLRP3* and *ASC* (*Pycard*) deficient mice were protected against obesity-induced metabolic dysfunction (Figure 3), these mice were also able to suppress development of hepatic steatosis during HFD.

### Obesity-Induced LV Remodeling Is Not Associated With Cardiac Fibrosis and Inflammation

**Figure 5:**
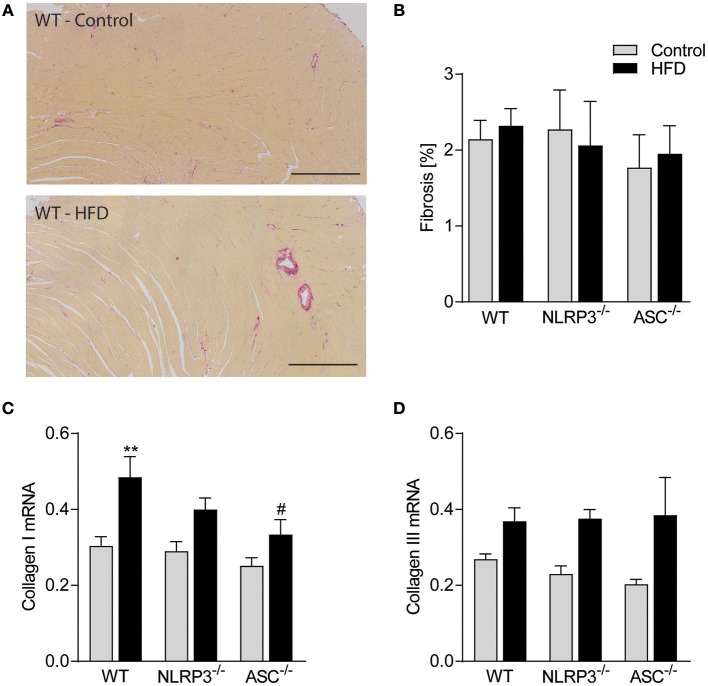
Obesity-induced LV remodeling is not associated with cardiac fibrosis. WT, *Nlrp3*^−/−^, and *Asc*^**−/−**^ (*Pycard*^−/−^) male mice were exposed to high fat diet (HFD; 60 cal% fat) or control diet for 52 weeks and cardiac fibrosis was evaluated. **(A)** Representative images of picrosirius red stained left ventricle (LV) from a WT mouse on control diet and HFD. Scale bar: 500 μm. **(B)** Quantification of picrosirius red positive areas in LV. LV expression of **(C)** collagen I mRNA and **(D)** collagen III mRNA. WT: Control, *n* = 10; HFD, *n* = 10, *Nlrp3*^−/−^: Control, *n* = 7; HFD, *n* = 7, and *Asc*^**−/−**^ (*Pycard*^−/−^): Control, *n* = 7; HFD, *n* = 7. Data are shown as mean ± SEM. ^**^P < 0.01 *vs*. control diet; ^#^*P* < 0.05 vs. WT-HFD as determined by two-way ANOVA and Tukey's multiple comparisons test.

**Figure 6:**
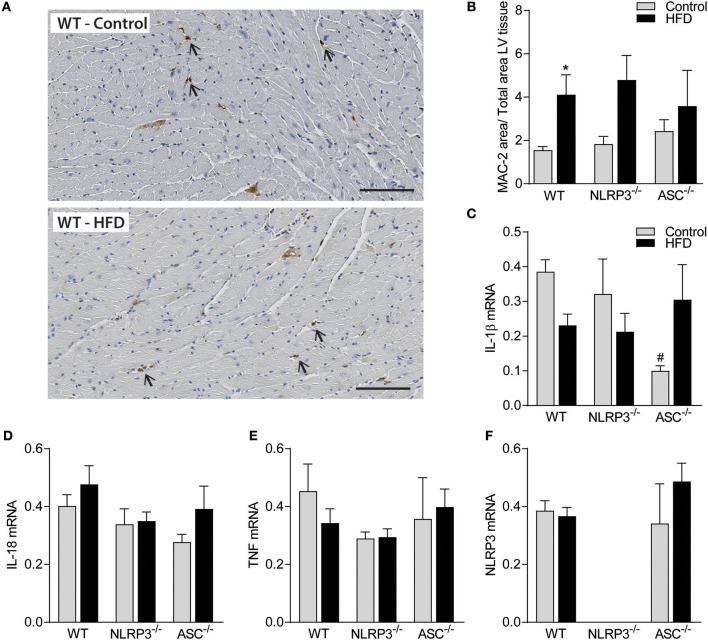
Obesity-induced LV remodeling is not associated with cardiac inflammation. WT, *Nlrp3*^−/−^, and *Asc*^**−/−**^ (*Pycard*^−/−^) male mice were exposed to high fat diet (HFD; 60 cal% fat) or control diet for 52 weeks and cardiac inflammation was evaluated. **(A)** Representative images of Mac-2 positive macrophages (arrows) in left ventricle (LV) from a WT mouse on control diet and HFD. Scale bar: 100 μm. **(B)** Quantification of Mac-2 positive areas in LV. LV gene expression, of **(C)** interleukin (IL)-1β mRNA, **(D)** IL-18, **(E)** tumor necrosis factor (TNF), and **(F)** NLRP3, presented relative to levels of GAPDH mRNA. WT: Control, *n* = 10; HFD, *n* = 10, *Nlrp3*^−/−^: Control, *n* = 7; HFD, *n* = 7, and *Asc*^**−/−**^ (*Pycard*^−/−^): Control, *n* = 7; HFD, *n* = 7. Data are shown as mean ± SEM. ^*^P < 0.05 vs. control diet; ^#^*P* < 0.05 vs. WT-HFD as determined by two-way ANOVA and Tukey's multiple comparisons test.

Previous studies have suggested that cardiac fibrosis and inflammation are potential contributors to obesity-induced changes in cardiac structure and function (29). However, as indicated in Figure 5A and quantified in Figure 5B, there was no cardiac fibrosis development in none of the obese mouse genotypes. Moreover, although we found increased gene expression of collagen I mRNA in obese WT mice, in general the differences were modest across the different genotypes and without any differences in collagen III mRNA levels (Figures 5C,D). Cardiac inflammation was evaluated by staining for Mac-2 positive macrophages (Figure 6A). We did observe a significant increase in Mac-2 positive staining in WT mice on HFD compared to WT mice on control diet (Figure 6B), with the same tendency in *Nlrp3*^−/−^ and *Asc*^**−/−**^ (*Pycard*^−/−^) mice. The absolute number of Mac-2 macrophages was however low in all genotypes. Moreover, cardiac gene expression of IL-1β, IL-18, and TNF was not statistically different between mice on control diet and HFD and between the different genotypes (Figures 6C–E). We also evaluated cardiac NLRP3 gene expression, and in line with expression of inflammatory cytokines, there was no increase in NLRP3 mRNA in WT and *Asc*^**−/−**^ (*Pycard*^−/−^) mice on HFD (Figure 6F). Finally, we were not able to detect the mature IL-1β protein by western blot technique within the myocardium in either of the genotypes (data not shown). These results may suggest that HFD-induced myocardial remodeling was not related to local cardiac inflammation or fibrosis, but rather systemic effects, i.e., inflammation and metabolic changes.

### Cardiac Insulin Sensitivity Is Preserved in Obese *NLRP3* Deficient Mice

**Figure 7:**
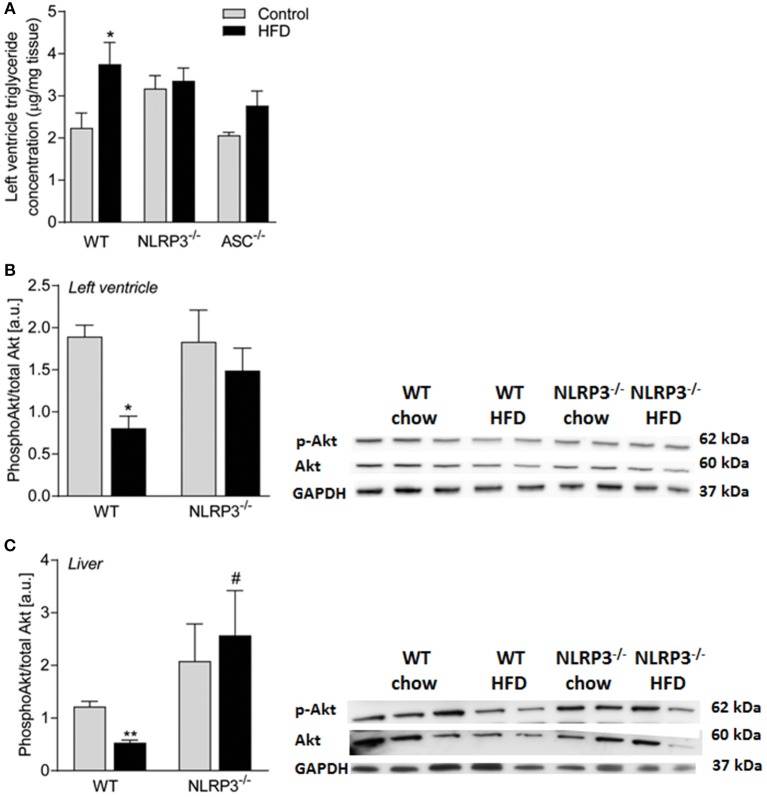
Cardiac insulin sensitivity is preserved in obese NLRP3 deficient mice. WT, and *Nlrp3*^−/−^ male mice were exposed to high fat diet (HFD; 60 cal% fat) or control diet for 52 weeks. **(A)** Left ventricle concentration of triglycerides [WT: Control, *n* = 10; HFD, *n* = 10, *Nlrp3*^−/−^: Control, *n* = 7; HFD, *n* = 7, and, *Asc*^**−/−**^ (*Pycard*^−/−^): Control, *n* = 7; HFD, *n* = 7]. WT and *Nlrp3*^−/−^ mice on control diet or HFD for 52 weeks were fasted for 4 h and received an i.p. injection of insulin (2 IU/kg). Mice were euthanized and after 10 min heart and liver were extirpated. Ratio of phosphorylated (Ser473) Akt to total Akt in mice on control diet or HFD in **(B)** left ventricle and **(C)** liver (WT: Control, *n* = 6; HFD, *n* = 4, *Nlrp3*^−/−^: Control, *n* = 4; HFD, *n* = 4) were determined by immunoblot analysis. Data are normalized to corresponding GAPDH. Data are shown as mean ± SEM. ^*^P < 0.05, ^**^P < 0.01 vs. control diet; ^#^*P* < 0.05 vs. WT-HFD as determined by two-way ANOVA and Tukey's multiple comparisons test.

As in the livers (Figure 4), heart homogenates from WT mice showed a significant HFD-induced elevation of TG content, which was not seen in the *Nlrp3*^−/−^ and *Asc*^**−/−**^ (*Pycard*^−/−^) hearts (Figure 7A). It is increasingly accepted that impaired insulin signaling could affect metabolic changes in various tissues during obesity (30). To determine whether downstream insulin signaling was altered in the hearts and livers of obese WT and *Nlrp3*^−/−^ mice, we examined the acute effect of a subcutaneous injection of insulin (2 IU/kg) on phosphorylation at Ser473 of Akt, a major target of insulin receptor signaling. The serine-threonine kinase Akt is activated by several ligand-receptor systems previously shown to be cardioprotective (31). While obese WT mice showed a marked reduction in Akt phosphorylation upon insulin treatment in both cardiac and hepatic tissue, *Nlrp3*^−/−^ mice were protected against this obesity-induced effect (Figures 7B,C).

## Discussion

Obesity and metabolic disease-related cardiac remodeling and HF are a growing worldwide concern (4, 5). The NLRP3 inflammasome may represent a molecular link between over nutrition, metabolic stress, inflammation and development of metabolic and cardiovascular diseases. Herein, we examined the effect of long-term HFD consumption on obesity associated cardiac remodeling in *NLRP3* and *ASC* (*Pycard*) deficient mice. We found that the cardiac hypertrophic response to obesity was independent of the NLRP3 inflammasome, while deficiency of NLRP3 and ASC blunted the concentric form of cardiac remodeling and the impairment of diastolic function seen in obese WT mice. Whereas, NLRP3 and ASC deficiency attenuated systemic inflammation and metabolic disturbances in HFD fed mice; long-term HFD did not induce significant cardiac fibrosis or inflammation, suggesting that the beneficial effects of NLRP3 inflammasome deficiency on myocardial remodeling at least partly reflect systemic mechanisms. However, in both hepatic and cardiac tissue, NLRP3 inflammasome deficiency counteracted lipid accumulation and the impaired insulin signaling in WT mice on HFD. The latter mechanisms could be an important mediator of the beneficial effect of NLRP3 deficiency on HFD induced myocardial remodeling, linking systemic and local effects within the myocardium.

Many different models have been used to address mechanisms for how obesity and metabolic disease causes cardiac remodeling and HF development, including leptin or leptin receptor deficient mice and mice with pharmacologically induced (e.g., streptozotocin) diabetes (32). In this study we used a model of diet-induced obesity (60% calories from fat), as it recapitulates many of the obesity-associated conditions in humans, such as adipose tissue remodeling, insulin resistance and hepatic steatosis. The reported cardiac phenotype of mice fed a HFD over an extended period of time varies in severity (33). However, the phenotype of our obese mice is similar to some previous studies, i.e., not overt HF, but more resembling the early alterations associated with development of diabetic cardiomyopathy (34, 35). Thus, while others have observed HF developing after 15 weeks using the same protocol (36), we found consistent cardiac hypertrophy and a concentric form of LV remodeling without overt HF in the WT mice. Our data show that although *NLRP3* and *ASC* (*Pycard*) deficient mice gained significantly less weight after 1 year on a HFD, all three genotypes did develop obesity, which was associated with cardiac hypertrophy, with similar heart weights and cardiomyocyte cross-sectional areas in the different mouse strains. However, NLRP3 and *ASC* (*Pycard*) deficiency appears to be preventive against LV concentric remodeling with potentially beneficial effects on cardiac geometry and diastolic dysfunction, the latter may be of particular relevance in relation to metabolic induced cardiomyopathy (4).

Inflammation is suggested to play a pathogenic role in development of myocardial dysfunction, including diabetic cardiomyopathy and HF with preserved ejection fraction (HFpEF). Others and we have previously implicated the NLRP3 inflammasome as a pathogenic mediator acting locally in the heart in ischemia reperfusion injury (19, 37, 38). Somewhat surprisingly, we did not find increased cardiac inflammation or cardiac fibrosis after 52 weeks of HFD in the present study. However, this is in fact in line with some previous studies (34, 35), illustrating the contrast between our model and those of e.g., leptin receptor deficiency (i.e., *db/db* mice) and type 1 diabetes (e.g., streptozotocin treatment) (33). In a rat model of streptozotocin and HFD-induced diabetic cardiomyopathy with severe cardiac inflammation and fibrosis, Luo et al. previously reported beneficial effects of NLRP3 gene silencing (39). More recently, and in contrast to our findings, Pavillard et al. showed reduced cardiac hypertrophy in *NLRP3* deficient mice and NLRP3 inhibition with MCC950 during HFD for 15 weeks (40). This discrepancy might be explained by the different durations of the experiments, 15 and 52 weeks, respectively. Also, they did not include *in vivo* examination of cardiac structure and function. Nonetheless, in our opinion, long-term exposure to HFD is a relevant model for examining the effects of an unhealthy diet and obesity on the myocardium, and will more accurately reflect the situation in patients with moderate obesity, T2DM, liver steatosis, hyperlipidemia and insulin resistance than the models of leptin receptor deficiency (i.e., *db/db* mice) and type 1 diabetes (e.g., streptozotocin treatment).

NLRP3 inflammasome is considered to have a critical role in sensing obesity-associated metabolic stress and mediating the associated inflammatory response and insulin resistance development (14, 41). This was confirmed in our study with a marked reduction in systemic, hepatic and also cardiac measures of insulin resistance. It is increasingly accepted that impaired insulin signaling could affect metabolic changes in various tissues during obesity (30). Moreover, the serine-threonine kinase Akt, a major target of insulin receptor signaling, has previously shown to be cardioprotective. This is in line with our results showing that while obese WT mice exhibited a marked reduction in Akt phosphorylation upon insulin treatment in both cardiac and hepatic tissue, this was not seen in NLRP3^−/−^ mice. The latter mechanism could be an important mediator of the beneficial effect of NLRP3 deficiency on HFD induced myocardial remodeling, linking systemic and local effects within the myocardium.

Moreover, plasma levels of mature IL-18, activated by the NLRP3 inflammasome, and TNF, a cytokine that could be activated down-stream to NLRP3 activation, were increased with obesity and markedly reduced in inflammasome deficient mice. Thus, even though there was no cardiac inflammation, there was an NLRP3-dependent systemic inflammatory response to obesity. Also, NLRP3 inflammasome deficiency reduced triglyceride levels both in the liver and within the heart. Based on our own and previous findings, it is tempting to term the *Nlrp3*^−/−^ and *Asc*^**−/−**^ (*Pycard*^−/−^) mice as models of cardiac response in metabolically healthy obesity (42). In extension of this, we hypothesize that the hypertrophic response we observe is related to the obesity *per se*. However, the concentric remodeling, a hallmark feature of diabetic cardiomyopathy and HFpEF was not observed in the inflammasome deficient mice suggesting that these features may be NLRP3 inflammasome-dependent. The lack of local cardiac inflammation and fibrosis in our results, suggests that the cardiac remodeling and dysfunction in obese WT mice is more likely mediated by external factors, such as systemic inflammatory and metabolic responses, rather than intrinsic processes in the heart, and these systemic responses are attenuated in NLRP3 inflammasome deficient mice. It is, therefore, tempting to hypothesize that NLRP3 inflammasome may link low-grade systemic inflammation to maladaptive myocardial remodeling and impaired diastolic function with insulin resistance as an important mediator within the myocardium. Notably, this model is in agreement with the novel HFpEF paradigm postulated by Paulus and Tschope, which suggest that a systemic inflammatory state induced by comorbidities, such as obesity and diabetes mellitus, triggers concentric cardiac remodeling and LV dysfunction in HFpEF (6). Our findings may suggest that NLRP3 inflammasome could be an important mediator in this process. Whereas, we found significantly higher levels of the mature IL-18 protein in WT as compared to *Nlrp3*^−/−^ and *Asc*^**−/−**^ (*Pycard*^−/−^) mice, none of the genotypes showed detectable levels of IL-1β in plasma (multiplex) and myocardium (western blot). The inability to measure IL-1β does, however, not mean that this NLRP3 inflammasome product is of less importance than IL-18. It could rather reflect technical problems with measuring this cytokine in mice models that has also previously been recognized (43). Although being a very potent cytokine, IL-1β is usually released in small amounts and has a very short plasma half-life (44).

The present study has some limitations as we did not use co-housed littermate controls all through the study. To minimize the effects of other factors than genetics, including effects on gut microbiota, the separate mouse strains were littermates; bred from the same parents, raised in the same cage until weaning where 4–6 mice of the same strain where co-housed in the same open cages. Gut microbiota has been recently established to have a contributory role in the development of cardio-metabolic disorders, such as atherosclerosis, obesity, and T2DM (45), and the impact of this “new organ.” should be investigated in forthcoming studies. Moreover, based on our experimental approach, we cannot conclude if the cardiac phenotype in *Nlrp3*^−/−^ and *Asc*^**−/−**^ (*Pycard*^−/−^) mice is secondary to weight gain or caused by direct effect of NLRP3 inflammasome on myocardial function.

Collectively, our data suggests that obesity drives cardiac hypertrophy *per se*, while systemic inflammation and metabolic dysfunction promotes adverse effects on cardiac remodeling and function, and these systemic effects were attenuated in *Nlrp3*^−/−^ and *Asc*^**−/−**^ (*Pycard*^−/−^) mice. Although our data may suggest a role for improved insulin-mediated Akt phosphorylation in *Nlrp3*^−/−^ and *Asc*^**−/−**^ (*Pycard*^−/−^) mice, further studies are needed to elucidate the molecular mechanisms for the role of NLRP3 activation during HFD, including the effects on myocardial remodeling. Further studies should also address NLRP3 inflammasome as a target for therapy in both experimental and clinical metabolic induced cardiac remodeling including HFpEF.

## Ethics Statement

The experimental animal protocol (FOTS id 4641) was approved by the Norwegian Animal Research Committee and conforms to the Guide for the Care and Use of Laboratory Animals published by the US National Institutes of Health (NIH Publication, 8th Edition, 2011).

## Author Contributions

MS, PA, TR, and AY conceived and designed the research and drafted the manuscript. MS, IS, ML, KA, JA, LZ, SH, BB, JØ, MB, EL, RB, TR, and AY acquired the data. MS, TR, and AY performed statistical analysis. MS, IS, ML, KA, JA, LZ, SH, BB, JØ, MB, EL, RB, PA, TR, and AY made critical revision of the manuscript.

### Conflict of Interest Statement

The authors declare that the research was conducted in the absence of any commercial or financial relationships that could be construed as a potential conflict of interest.
