# Supplementary material for: NLRP3 Inflammasome Promotes Myocardial Remodeling During Diet-Induced Obesity
Source: Front Immunol. 2019 Jul 16;10:1621. doi: 10.3389/fimmu.2019.01621 (PMC6648799; doi:10.3389/fimmu.2019.01621)
Supplement: Supplementary file 1 [file Data_Sheet_1.docx]

M Sokolova *et al*. NLRP3 inflammasome promotes myocardial remodeling during diet-induced obesity.

**Supplementary results**

**Supplementary Table 1**

Sequences of the primers used for RT-PCR

|  | Species | Sequence (5’ 🡪 3’) | Acc.nr |
| --- | --- | --- | --- |
| IL-1β | Mouse | (+)-GCCACCTTTTGACAGTGATGAG | NM_008361 |
|  |  | (-)-GTTTGGAAGCAGCCCTTCATC |  |
| TNF | Mouse | (+)-AGACCCTCACACTCAGATCATCTTC | NM_013693 |
|  |  | (-)-CCACTTGGTGGTTTGCTACGA |  |
| GAPDH | Mouse | (+)-CCAAGGTCTCCATGACAACTT  (-)-AGGGGCCATCCACAGTCTT | NM_008084 |
| IL-18  NLRP3  Coll I  Coll II | Mouse  Mouse  Mouse  Mouse | (+)-AAGAACAAGATCATTTCCTTTGAGGA  (-)-GGAACACGTTTCTGAAAGAATATGAG  (+)-CATGTTGCCTGTTCTTCCAGAG  (-)-CGGTTGGTGCTTAGACTTGAGA  (+)-CCTGAGTCAGCAGATTGAGAACA  (-)-TCGATCCAGTACTCTCCGCTCT  (+)-TCTATGAATGGTGGTTTTCAGTTCA  (-)-TTTTTGCAGTGGTATGTAATGTTCT | NM_008360  NM_145827  NM_007742  NM_009930 |

**Supplementary Figure S1**

**NLRP3 inflammasome does not affect food intake.**

WT, *Nlrp3*^−/−^, and *Asc*^−/−^ (*Pycard*^−/−^) male mice were exposed to high fat diet (HFD; 60 cal% fat) or control diet. Food intake was assessed at 21 weeks of age. WT: Control, *n*=7; HFD, *n*=7, *Nlrp3*^−/−^: Control, *n*=7; HFD, *n*=7, and *Asc*^−/−^: Control, *n*=7; HFD, *n*=7. Data are shown as mean ± SEM.

**Supplementary Figure S2**

**NLRP3 inflammasome does not affect left ventricular gene expression of atrial natriuretic peptide with obesity.**

WT, *Nlrp3*^−/−^, and *Asc*^−/−^ (*Pycard*^−/−^) male mice were exposed to high fat diet (HFD; 60 cal% fat) or control diet for 52 weeks and left ventricular gene expression of atrial natriuretic peptide (ANP) was evaluated. WT: Control, *n*=10; HFD, *n*=10, *Nlrp3*^−/−^: Control, *n*=7; HFD, *n*=7, and *Asc*^−/−^: Control, *n*=7; HFD, *n*=7. Data are shown as mean ± SEM.
